# Refrigeration of eggs influences the virulence of *Salmonella* Typhimurium

**DOI:** 10.1038/s41598-021-97135-4

**Published:** 2021-09-09

**Authors:** Samiullah Khan, Andrea R. McWhorter, Talia S. Moyle, Kapil K. Chousalkar

**Affiliations:** School of Animal and Veterinary Sciences, The University of Adelaide, Roseworthy, SA 5371 Australia

**Keywords:** Applied microbiology, Pathogens

## Abstract

*Salmonella* Typhimurium is a human pathogen associated with eggs and egg-derived products. In Australia, it is recommended that eggs should be refrigerated to prevent condensation that can enhance bacterial penetration across the eggshell. Except for the United States, the guidelines on egg refrigeration are not prescriptive. In the current study, in-vitro and in-vivo experiments were conducted to understand the role of egg storage temperatures (refrigerated vs ambient) on bacterial load and the virulence genes expression of *Salmonella* Typhimurium. The in-vitro egg study showed that the load of *Salmonella* Typhimurium significantly increased in yolk and albumen stored at 25 °C. The gene expression study showed that *ompR*, *misL*, *pefA*, *spvA*, *shdA*, *bapA*, and *csgB* were significantly up-regulated in the egg yolk stored at 5 °C and 25 °C for 96 h; however, an in-vivo study revealed that mice infected with egg yolk stored at 25 °C, developed salmonellosis from day 3 post-infection (p.i.). Mice fed with inoculated egg yolk, albumen, or eggshell wash stored at refrigerated temperature did not show signs of salmonellosis during the period of the experiment. Data obtained in this study highlighted the importance of egg refrigeration in terms of improving product safety.

## Introduction

Egg associated human salmonellosis is a significant economic burden on global public health systems^1–3^. It is generally recommended that eggs should be refrigerated to prevent condensation and subsequent bacterial penetration across the eggshell; however, condensation has not been significantly linked with *Salmonella* Enteritidis penetration level^4^. Guidelines for egg storage have been mentioned in documents, such as FAO guide^5^, HACCP and ISSO 22000^6^, UNECE Standard Egg-1, Chinese National Standard GB 2749–2015; however, it is not compulsory to refrigerate eggs in Australia. In Europe, the EC Regulation No. 589/2008 prevents eggs from refrigeration before sale to consumers^7^. In several countries, including Australia, table eggs are commonly stored at ambient temperature on supermarket shelves. Consequently, there is a continuing debate on whether to refrigerate or not refrigerate eggs. The principle justification for eggs refrigeration is to restrict the growth of pathogenic organisms within the edible components.

Eggs can be contaminated with *Salmonella* through vertical and horizontal routes during egg formation in the layer hen or handling in the supply chain. In Australia, *Salmonella* Typhimurium is the major food-borne pathogen of human salmonellosis, which is often associated with the consumption of contaminated eggs or egg-based products^2^. However, other serotypes such as *S.* Infantis and *S.* Enteritidis also have been implicated^8,9^. Washing is an effective strategy for controlling *Salmonella* on table eggs, but it can cause damage to the egg cuticle^8^. Once *Salmonella* enters the egg internal contents, it can replicate at ambient temperature^10,11^. In many countries, including Australia, supermarkets and grocery shops have no prescriptive guidelines on egg storage. Therefore, it is important to understand the role of storage temperatures on survivability and growth of *Salmonella* Typhimurium in eggs and its subsequent virulence factors in causing salmonellosis in humans. Relevant research on the behaviour of *Salmonella* in eggs about storage temperatures were either focused on the penetration of bacteria across the eggshell^12,13^ or using mainly *Salmonella* Enteritidis^10,13^ as a model organism. The bacterial load on the eggshell surface is inversely affected by storage time and temperature, while an increase in relative humidity supports the bacterial survivability as shown for *Salmonella* Enteritidis^13^. The survival behaviour of *Salmonella* Enteritidis both in an egg^14^ and culture media^15^ is different from other serotypes including *Salmonella* Typhimurium. Therefore, studies are required to understand the behaviour of *Salmonella* Typhimurium in eggs and the subsequent development of salmonellosis through the consumption of the contaminated egg components.

In the host (as shown for mouse), upon ingestion, *Salmonella* Typhimurium survives in the acidic environment of the stomach and migrates to the intestine^16^, where it invades the intestinal epithelia and triggers inflammation and onset of clinical disease^17^. *Salmonella* uses Type III secretory systems encoded by SPI (*Salmonella* pathogenicity island) 1 and SPI2 for epithelial cell invasion and survival^17^. A previous study has shown that an inoculum of less than 10 colony forming units (CFUs) of *Salmonella* Typhimurium is sufficient to cause clinical salmonellosis in healthy individuals^18^. Also in field conditions, the load of *Salmonella* on egg surface and in shed environment has shown to be variable^19^. Therefore, in the current study, the inoculum dose for *Salmonella* Typhimurium grown in egg components was not adjusted for mice infection.

Various studies have investigated the effects of storage temperatures on the growth and survivability of *Salmonella* in eggs^10,11,20^; however, no subsequent investigations on the development of salmonellosis through the consumption of the contaminated egg contents have been conducted. The growth kinetics of *Salmonella* Typhimurium is regulated by temperature. For example, the survivability of *Salmonella* Typhimurium on eggshell is better at 22 °C, while bacterial load decreases with storage time at 4 °C^21^. *Salmonella* Typhimurium grows well in the yolk at either 15 °C or 22 °C, whereas its growth in albumen is temperature dependent^21^. A study has linked the up-regulation of *yafD* and *xthA* with the *Salmonella* Enteritidis survival in albumen^22^.

*Salmonella* serotypes specific differences in survivability in egg albumen^14,23^ suggest that *Salmonella* Typhimurium may regulate its transcriptional machinery differently. To answer this question, we hypothesized that the virulence of *Salmonella* Typhimurium is enhanced at 25 °C compared with refrigeration temperature and, therefore, if consumed, contaminated eggs stored at ambient temperature will cause clinical disease in mice. The present study had three main objectives: (1) Understand the growth and survivability of *Salmonella* Typhimurium in eggs stored at 5 °C and 25 °C. (2) Determine the effects of storage temperatures on *Salmonella* Typhimurium gene expression in yolk, albumen and on the eggshell surface. (3) Study the virulence of *Salmonella* Typhimurium cultured in raw egg components in BALB/c mice.

## Methods

### *Salmonella* growth and survivability kinetics in eggs at different temperatures

#### Eggs preparation and inoculation with Salmonella Typhimurium

For both the in-vitro and in-vivo experiments, a pure culture of *Salmonella* Typhimurium definitive type 9 stored at − 80 °C in 50% glycerol was revived on nutrient agar (NA; ThermoFisher Scientific, Australia) and subcultured in Luria Bertani (LB; ThermoFisher Scientific, Australia) broth until the optical density (OD) at 600 nm reached approximately 1. Fresh eggs directly obtained from a 33-week old cage layer hen flock were sanitized by dipping in 75% ethanol for 90 s and air dried completely in a class II biosafety cabinet. To study the survivability of bacteria on the eggshell surface, individual eggs (*n* = 6 in each treatment group) were dipped for 90 s in 1 × 10^6^ CFU/mL of *Salmonella* suspension prepared in LB broth. To confirm bacterial deposition on the eggshell surface, an extra set of eggs (*n* = 6) were dipped in the inoculum and processed immediately for the recovery of *Salmonella*. For studying *Salmonella* growth or survivability in the albumen and yolk, individual eggs received a 0.1 mL inoculum of 1 × 10^3^ CFU per egg directly injected into the egg albumen or yolk and the injection holes were sealed. This dose was selected to mimic a field scenario where a low load (1.71 log_10_ MPN per egg) of *Salmonella* on an egg has been reported^19^. All the treatment groups were incubated at 5 °C or 25 °C for either 96 h or 28 days. The 5 °C treatment eggs were put in a 55 L plastic container and stored in a cool room and the 25 °C condition eggs were stored in an incubator. Two separate hygrometers were used to monitor the relative humidity (%) levels of the two treatment groups. These two temperature conditions were selected as most likely during storage eggs are exposed to an ambient temperature close to 25 °C. The control eggs in each treatment group (*n* = 4 per treatment) were treated in sterile LB and processed in the same way as of the *Salmonella* inoculated eggs.

#### Quantitative recovery of Salmonella from the inoculated eggs

For estimation of *Salmonella* survivability on the eggshell surface, individual eggs from both the treatment groups (5 °C and 25 °C) were washed for 2 min in 10 mL of buffered peptone water (BPW; ThermoFisher Scientific, Australia) in Whirl–Pak sample bags, the rinsate was serially diluted (10 times) in phosphate buffered saline (PBS) and plated onto xylose lysine deoxycholate (XLD; ThermoFisher Scientific, Australia) agar plates for overnight incubation at 37 °C. *Salmonella* culturability in albumen and yolk of the stored eggs was also determined. Eggs were opened into a sterile 90 mm petri dishes, then poured into sterile bags for thorough mixing, and using a syringe, 0.1 mL of either albumen or yolk was added to 0.9 mL of BPW and mixed. Serial tenfold dilutions were prepared in PBS and plated onto XLD agar plates. The plates were incubated overnight at 37 °C and *Salmonella* colonies were enumerated to determine the total number of bacteria in each sample. The egg contents of the 5 °C stored samples were enriched for the qualitative assessment of *Salmonella* following a previously described method^19^. Briefly, 1 mL samples of yolk or albumen of individual eggs were enriched in 9 mL of BPW and incubated overnight at 37 °C. From the incubated samples, 100 μL was added to 10 mL of Rappaport–Vassiliadis soya peptone (ThermoFisher Scientific, Australia) broth and incubated overnight at 42 °C. The samples were streaked onto XLD, incubated overnight at 37 °C and read as positive or negative for characteristic *Salmonella* colonies. To rule out the chances of external contamination, control groups were processed in the same manner. The growth of *Salmonella* was recorded as CFU/mL of albumen or yolk or eggshell surface rinsate. The in-vitro egg experiments were repeated for a total of three times.

### Role of temperature and storage time in regulation of genes of *Salmonella* in egg components

To obtain good quality RNA from *Salmonella* inoculated egg components, albumen and yolk were inoculated with 1 × 10^8^ CFU/mL of *Salmonella*, while for the eggshell surface inoculation, intact individual eggs were dipped for 90 s in BPW (prepared in 1 L sterile beaker) that contained 1 × 10^9^ CFU/mL of *Salmonella*. Based on the protocol of TRIzol Reagent (Invitrogen, Australia), at least 1 × 10^7^ cells of bacterial origin are required to extract sufficient RNA. Our pilot experiment showed that 1 × 10^6^ CFUs of *Salmonella* per mL of egg content was not sufficient for quality RNA extraction.

#### Preparation of egg components

Intact eggs were sanitized in ethanol and allowed to dry thoroughly as described previously. As the volume of albumen and yolk vary from egg to egg; therefore, for precise measurement of per mL volume for *Salmonella* inoculation, egg contents were separated in sterile petri dishes. Maximum albumen (both the colloidal and watery parts) or yolk from individual eggs was aspirated using separate syringes and decanted into 50 mL Falcon tubes. The quantity of albumen or yolk in the tubes was adjusted based on *Salmonella* inoculum (1 × 10^8^ CFU/mL of yolk or albumen). There were 3 biological replicates for each of the egg components.

#### Preparation of Salmonella inoculum

A stock culture of *Salmonella* Typhimurium was revived on NA media plate and a single colony was subcultured in LB broth until the OD (measured at 600 nm) of the culture reached approximately 1. The inoculum dose for the egg surface was prepared in LB, while for the egg internal contents, *Salmonella* culture was pelleted by centrifugation (4000 × *g* for 15 min). The pellet was resuspended in 1 mL of PBS and its OD at 600 nm was read as described earlier. The culture was diluted to obtain 1 × 10^9^ CFU/mL of *Salmonella* and a 0.1 mL of this inoculum was added into either 0.9 mL of yolk or albumen. Thus, the final dose of *Salmonella* was 1 × 10^8^ CFU/mL of egg content. For the positive control group, *Salmonella* Typhimurium was incubated (with 3 biological replicates) in a shaking incubator at 37 °C in LB broth for 12 h and 1 × 10^8^ CFU/mL was processed for RNA extraction.

#### *Salmonella* RNA extraction from egg components and pure culture of bacteria

To recover *Salmonella* from the egg surface for RNA extraction, individual eggs (*n* = 3) at each incubation time-point (12, 72, and 96 h) from each treatment group (5 °C and 25 °C) were rinsed for 2 min in 5 mL BPW. The rinsate was centrifuged at 5000 × *g* for 10 min. The bacterial pellet was resuspended in 0.25 mL of PBS into which 0.75 mL of TRIzol Reagent was added and maintained on ice. To recover *Salmonella* from egg contents, 1 mL of albumen or yolk (*n* = 3) at each incubation time-point (12, 72 and 96 h) and treatment group (5 °C and 25 °C) was solubilized in 1 mL of sodium citrate (0.2 M, pH unadjusted), vortexed and centrifuged at 18,000 × *g* for 5 min at 4 °C. The supernatant was discarded, and the bacterial pellet was resuspended in 0.25 mL of PBS into which 0.75 mL of TRIZol Reagent was added and the samples were maintained on ice. For the extraction of bacterial RNA from pure culture, 1 mL of *Salmonella* inoculated LB broth (1 × 10^8^ CFU/mL) was centrifuged at 18,000 × *g* for 5 min at 4 °C, the pellet was resuspended in 0.25 mL of PBS into which 0.75 mL of TRIZol Reagent was added and the samples were maintained on ice until processed for RNA extraction. The TRIZol Reagent added samples were briefly homogenised using a hand-held IKA T10 Basic Ultra Turrax Homogenizer (Wilmington, NC, USA) while maintaining the tubes on ice in 20 mL container. The homogenised samples were incubated for 7 min on ice and 0.1 mL of 1-Bromo-3-chloropropane (BCP; Sigma, Australia) was added into each sample. The samples were incubated for 3 min at room temperature and centrifuged at 12,000 × *g* for 15 min at 4 °C to separate RNA from DNA and protein. Maintaining the samples on ice, from the top aqueous layer, 0.5 mL was transferred into new tubes and the process for RNA extraction was completed as per the manufacturer’s instructions. The quality and purity were tested in Nanodrop 1000 and the RNA was stored at − 80 °C until used for cDNA synthesis and Fluidigm PCR.

#### Primer, cDNA synthesis and Fluidigm PCR

A total of 95 primers for targeted genes of *Salmonella* Typhimurium involved in stress response, virulence, survivability, metabolism, and host colonization were designed using NCBI as described in our previous publications^24,25^ and were synthesized by Merck, Australia. The main aim behind selecting these genes was to understand their regulations in egg components about storage temperature and their subsequent role in causing clinical salmonellosis in mice. Using PCR (see Supplementary material Text S1) and 2% agarose gel electrophoresis, all the primers were tested for target specificity in cDNA synthesized from *Salmonella* Typhimurium extracted RNA. To cross check the issue of genomic DNA contamination, RNA samples were also included in the PCR run. cDNA was synthesized from the extracted RNA as per the protocol of the QuantiTect Reverse Transcription Kit (Qiagen, Australia). Briefly, 1 μg of RNA per sample was subjected to genomic DNA removal in a 14 μL total reaction volume for 3 min at 42 °C as per the protocol of the kit. For the reverse transcription step, the samples (in 20 μL reaction volume) were incubated for 25 min at 42 °C and to inactivate the Quantiscript reverse transcriptase, a final incubation step for 3 min at 95 °C was executed. The cDNA samples were stored at − 80 °C until used for downstream analysis. A Fluidigm PCR was performed on all the cDNA samples using 95 genes in a 96.96 DYNAMIC ARRAY IFCS at the Australian Centre for Cancer Biology, Adelaide (Supplemental material Text S2).

The quantification cycle (Cq) values obtained were analyzed for relative fold change expression (2^−ΔΔCq^) using the data of *Salmonella* Typhimurium grown in LB broth for 12 h as a reference control and *rrsG (16S rRNA)* as a reference gene for data normalization. The *rrsG* was selected as a reference gene based on its least standard deviation value in the current experimental conditions. For a clear interpretation of up- or down-regulated genes, the relative expression data were presented as log_2_ fold change.

### Effect of *Salmonella* inoculated egg components on clinical disease in mice

#### Animal ethics

The experimental protocol was approved by the Animal Ethics Committee at The University of Adelaide under approval number S-2018-009. Specific pathogen free 6–8 week-old, female BALB/c mice were sourced from the Laboratory Animal Services at The University of Adelaide. The mice were fed ad libitum a commercial diet free from *Salmonella* following the guidelines specified in the “Australian Code for the care and use of animals for scientific purposes, 8th edition (2013).”

#### Ethics approval and consent to participate

The University of Adelaide, Animal Ethics Committee under Approval Number No., approved the experimental setup. S-2018-009. All animal experiments complied with the ARRIVE guidelines and were also carried out in accordance with the guidelines -specified in Australian Code for the Care and Use of Animals for Scientific Purposes 8th edition 2013.

#### Mice inoculation with egg components containing Salmonella

Mice were divided into 16 treatment groups with 7 mice per group (Table 1). Yolk, albumen, and eggshell wash used as inoculums were prepared as described for the in-vitro studies above. Briefly, the inoculated eggs were stored at either 5 °C or 25 °C for 96 h and individual mice received through oral gavage 0.1 mL of shell wash, yolk, or albumen that had either been inoculated or non-inoculated with *Salmonella* Typhimurium. The positive control groups received through oral gavage 1 × 10^3^ CFU/ 0.1 mL of *Salmonella* Typhimurium in LB broth stored at either 5 °C or 25 °C (inoculum adjusted) for 12 h. Further detail of the inoculum that individual mice in each treatment group received is outlined in Table 1.

**Table 1:** Treatment groups and inoculum dose used in the mice study.

Treatment	Inoculum
Negative control 5 °C	LB broth
Negative control 25 °C	LB broth
Shell wash control 5 °C	BPW
Albumen control 5 °C	Albumen
Yolk control 5 °C	Yolk
Shell wash control 25 °C	BPW
Albumen control 25 °C	Albumen
Yolk control 25 °C	Yolk
Positive control *Salmonella* in LB broth stored at 5 °C	10^3^ CFU
*Salmonella* from shell wash stored at 5 °C	10^1^ CFU
*Salmonella* in albumen stored at 5 °C	1 CFU
*Salmonella* in yolk stored at 5 °C	10^1^ CFU
Positive control *Salmonella* in LB broth stored at 25 °C	10^3^ CFU
*Salmonella* from shell wash stored at 25 °C	10^1^ CFU
*Salmonella* in albumen stored at 25 °C	10^2^ CFU
*Salmonella* in yolk stored at 25 °C	10^4^ CFU

The leftover inoculums from the treatment groups were maintained on ice, serially diluted and plated on XLD agar to confirm the actual dose of *Salmonella* Typhimurium that mice received. The experiment was conducted over 21 days. During the post-infection period (day 0 to 21), the mice were routinely observed for clinical signs of non-typhoidal salmonellosis (e.g. lethargy, hunching, ruffled fur) and mortality. Adhering to the Animal Ethics Committee Guidelines, mice suffering from the clinical disease with a score of five or above were humanely euthanized using carbon dioxide and the collected organs were processed for the quantification of *Salmonella*.

#### Salmonella detection in mice feces

From all the treatment groups, fecal samples were collected on day 3, 6, 9, 12, 15, and 18 post- infection and processed for *Salmonella* isolation. The cage and bedding materials were changed at every fecal sampling to minimize the risk of carryover. For the qualitative assessment of *Salmonella*, 1 g of fecal samples was mixed into 9 mL of BPW, vortexed and incubated overnight at 37 °C. The samples were processed following the RVS enrichment method previously described^19^. The plates were then examined to determine if the samples were positive (scored as 1) or negative (scored as 0) for *Salmonella* Typhimurium.

#### Salmonella load in organs

At the point of cull, segments of liver, spleen, ileum and cecum were collected and homogenized in tubes containing 0.5 mL of 0.9% saline and stainless-steel beads (2–8 mm). Homogenates were serially (tenfold) diluted and processed for the quantitative and qualitative assessment of *Salmonella*. For the quantitative assessment, 0.1 mL of the homogenate was directly plated onto XLD, incubated overnight at 37 °C and colonies were counted. The CFU data were expressed as log_10_ load of *Salmonella* per gram of the organ.

### Statistical analysis

The *Salmonella* load data were analyzed in GraphPad Prism version 8.0 using one- and two-way ANOVA with Tukey’s multiple comparison test for determining the level of significance (P < 0.05). The relative gene expression fold change was calculated by 2^^−ΔΔCq^ method and LB 12 h was used as a reference control. The log_2_ fold change data between the 12, 72 and 96 h were analyzed in StatView version 5.0.1 by repeated measure analysis and the level of significance was determined by PLSD (P < 0.05).

## Results

### Growth kinetics and survivability of *Salmonella* in egg components

**Figure 1:**
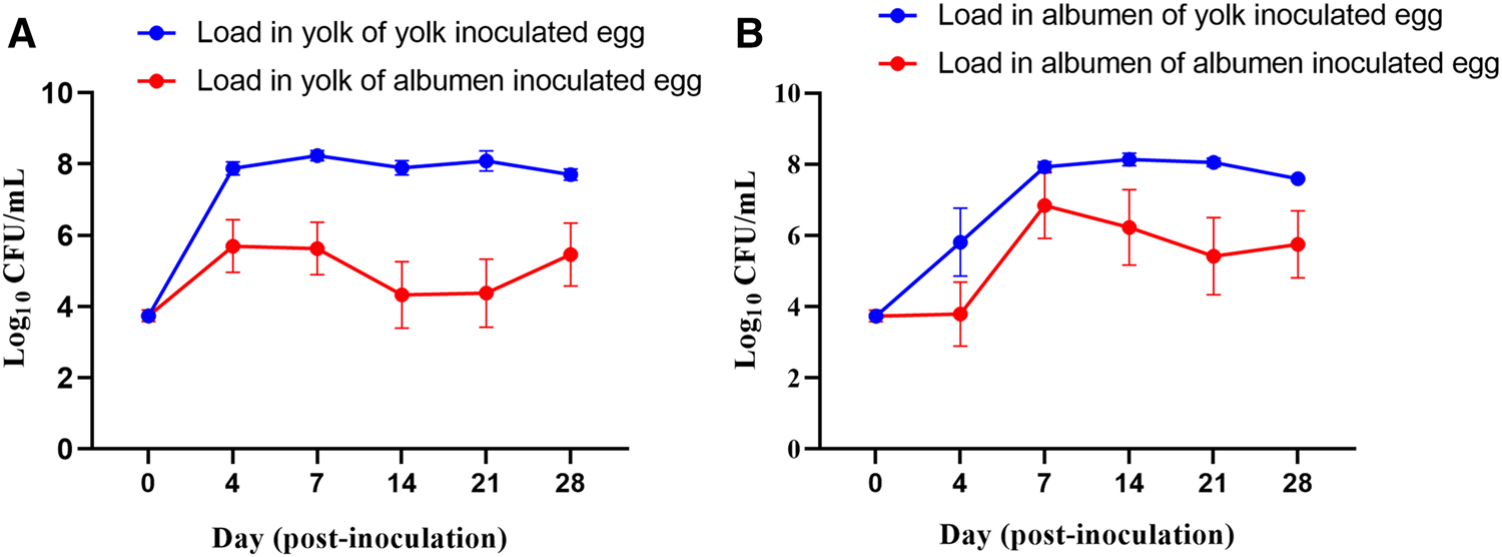
Load of *Salmonella* Typhimurium in yolk or albumen inoculated eggs stored at 25 °C. (**A**) Load in yolk of yolk and albumen inoculated eggs. (**B**) Load in albumen of yolk and albumen inoculated eggs. At each sampling time-point, 3 eggs from each treatment group were processed for the quantification of *Salmonella* Typhimurium load through direct plating on XLD media plates. At day 0, each egg was inoculated with 10^3^ CFU/0.1 mL of *Salmonella* Typhimurium directly injected to either yolk or albumen.

Post-inoculation with *Salmonella*, the relative humidity at both 5 °C and 25 °C varied from 76 to 82% over the 96 h egg storage experiment. The *in-vitro* experiment showed that *Salmonella* Typhimurium inoculated into egg yolk grew significantly to log_10_ 7.88 CFU/mL at day 4 of storage at 25 °C, whereas its growth plateaued from day 4 until day 28 of storage (Fig. 1A). This showed that after 96 h of storage, the load of *Salmonella* Typhimurium increased by log_10_ 3.88 CFU/mL in the yolk. The data showed that when inoculated in egg albumen, by day 4 post-inoculation, *Salmonella* Typhimurium migrated from albumen and grew to log_10_ 5.70 CFU/mL in the yolk (Fig. 1A). Load of *Salmonella* Typhimurium in the albumen of albumen-inoculated eggs remained constant from day 0 to day 4 of storage and then increased to log_10_ 6.84 CFU/mL (Fig. 1B). Overall, these data showed that the growth of *Salmonella* Typhimurium significantly increased in the yolk within 96 h of storage at 25 °C of the inoculated eggs. In the qualitative assessment, 75.6% of the yolk and 15.6% of the albumen samples of the eggs stored at 5 °C were positive for *Salmonella* after the enrichment process (Supplementary Fig. S1A), demonstrating that *Salmonella* Typhimurium survived better in yolk than albumen over the 28 days of storage (Supplementary Fig. S1B).

**Figure 2:**
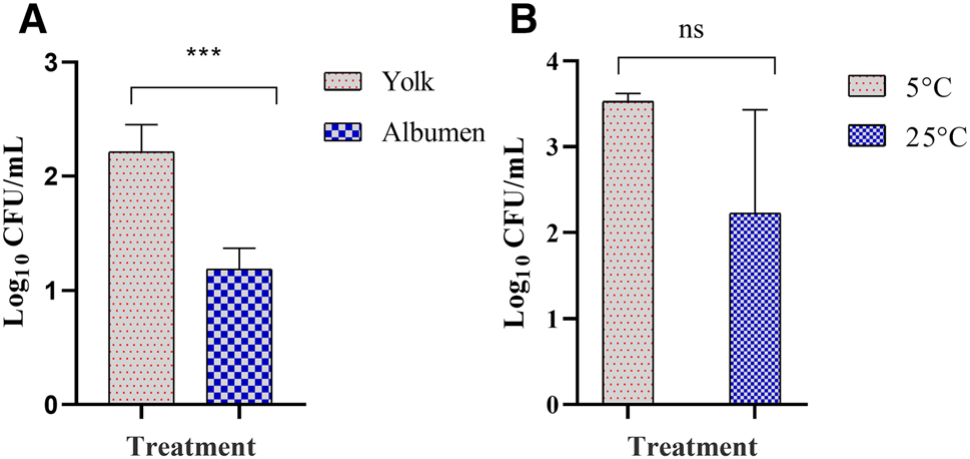
Survivability of *Salmonella* Typhimurium in eggs stored for 96 h. (**A**) Yolk and albumen inoculated eggs stored at 5 °C. (**B**) Eggshell inoculated eggs stored at 5 °C or 25 °C. Individual eggs in each of the yolk and albumen treatment groups received 1 × 10^3^ CFU/0.1 mL, and the eggs were stored at 5 °C for 96 h. For the shell treatment groups, intact eggs were dipped in *Salmonella* inoculum containing 1 × 10^6^ CFU/mL of LB broth and stored at 5 °C or 25 °C for 96 h. The stored eggs were processed for the recovery of *Salmonella* and the load was presented as log_10_ CFU/ mL of albumen, yolk or shell rinsate. Within each storage temperature, different superscripts show significant differences (P < 0.05). Values are mean of log_10_ CFU ± S.D.

As the samples stored at 5 °C did not show growth of *Salmonella* after the direct plating; therefore, in the subsequent in-vitro experiments, inoculated eggs were stored only for 96 h. In contrast to 25 °C, *Salmonella* Typhimurium counts remained constant for yolk and albumen samples stored at 5 °C for 96 h (Fig. 2A). However, the load of *Salmonella* Typhimurium was significantly higher in the yolk compared with the albumen. *Salmonella* survived numerically higher on the eggshell surface stored at 5 °C compared with the 25 °C (Fig. 2B). For the eggshell treatment groups, on average, each egg had 9.7 × 10^5^ (or 5.99 on log_10_ scale) CFU of *Salmonella*, which was just under 1 mL of inoculum that contained 1 × 10^6^ CFU. Therefore, the survivability of *Salmonella* on the eggshell surface stored at 5 °C for 96 h was 3.53 CFU/mL (on a log_10_ scale) that showed a 0.46 log reduction (Fig. 2B). The survivability of *Salmonella* on the eggshell surface stored at 25 °C for 96 h was 2.22 CFU/mL that showed a 1.76 log reduction (Fig. 2B). Overall, the eggshell data showed that *Salmonella* Typhimurium survived numerically better at 5 °C compared with the 25 °C storage temperature.

### *Salmonella* transcription profile is affected by egg storage temperature

To understand the effects of storage temperatures on the gene expression of *Salmonella* Typhimurium in the yolk, albumen and on eggshell, Fluidigm PCR was performed on *Salmonella* RNA extracted from inoculated egg contents whereas RNA obtained from *Salmonella* grown in LB for 12 h at 37 °C acted as a control for the relative gene expression analysis. Overall, several genes were up- or down-regulated in different egg components and storage temperatures; therefore, only the genes significantly up-regulated at least at one storage time point or involved in pathways that include *Salmonella* pathogenicity islands are presented in this manuscript. Many of the key *Salmonella* genes involved in virulence and colonization in the mammal host were downregulated in albumen and on egg surface of the samples that were stored at 5 °C and 25 °C.

#### Stress response, host colonization, virulence and invasion

**Figure 3:**
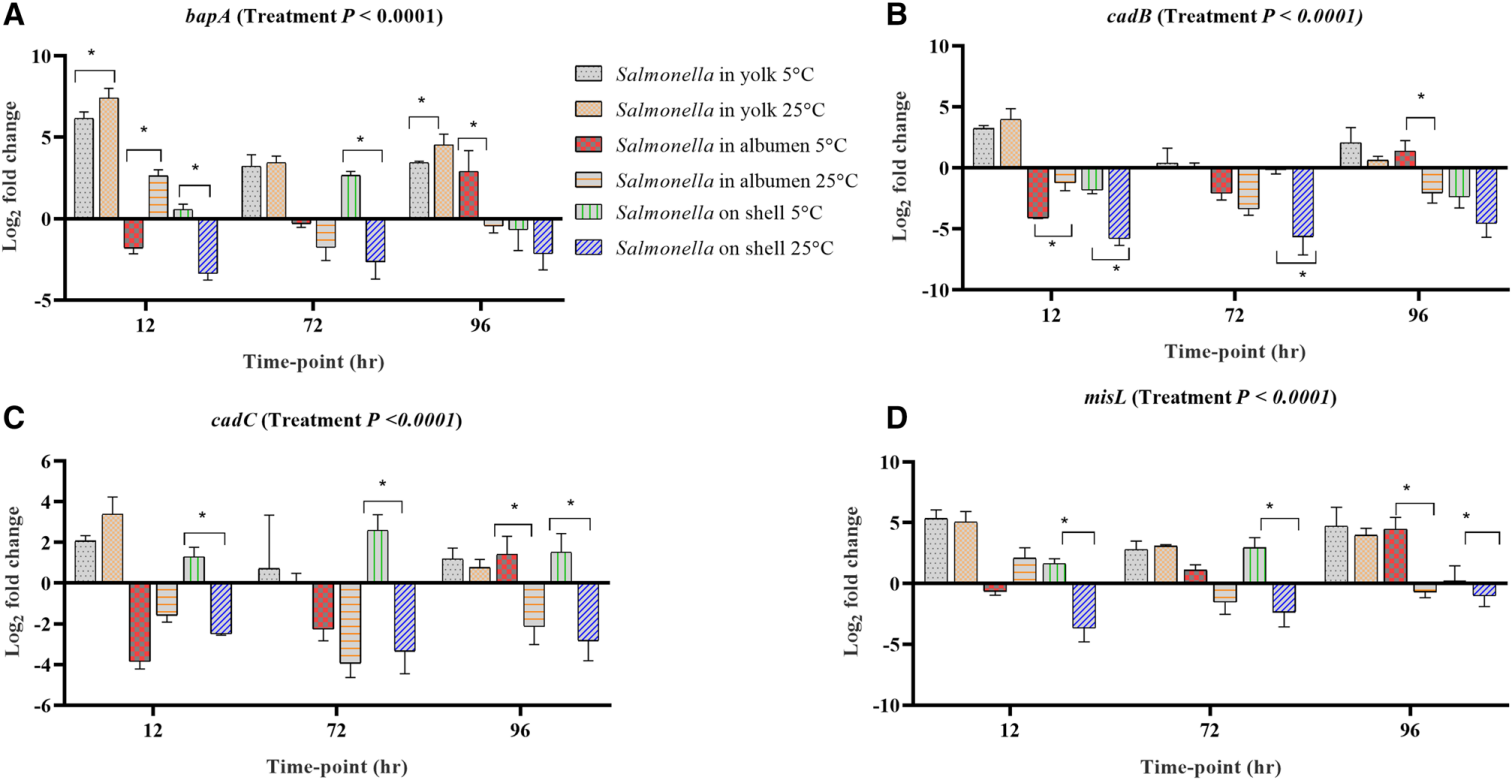
Regulation of genes in *Salmonella* Typhimurium inoculated into yolk, albumen and eggshell stored at 5 °C and 25 °C. Relative expression (log_2_ fold change ± S.E.) of (**A**) *bapA*; (**B**) *cadB*; (**C**) *cadC* and (**D**) *misL* at 12, 72 and 96 h of incubation time. Within each egg component and storage time-point, asterisks across the bars show significant differences (P < 0.05).

Among the genes involved in host colonization, aggregation and infection, the expression levels of *bapA*, *cadB*, *cadC*, *misL*, *ompR*, *shdA*, *spvA* and *spvC* in at least one egg content and storage time-points were up-regulated (Fig. 3). Among them, *bapA* is involved in biofilm formation and host colonization, and the deletion of *bapA* has shown reduced colonization potential in the gut of mice^26^. *bapA* was significantly up-regulated in the yolk and its mean expression was significantly higher in the 25 °C stored yolk compared with the 5 °C (Fig. 3A). However, in albumen and on the egg surface, the expression of *bapA* was not consistent, where it was down-regulated on the egg surface of the samples stored at 25 °C. *cadB*, *cadC* and *misL* were up-regulated in the yolk stored both at 5 °C and 25 °C, while in albumen, these genes were up-regulated at 96 h of incubation in the samples stored at 5 °C (Fig. 3B–D).

**Figure 4:**
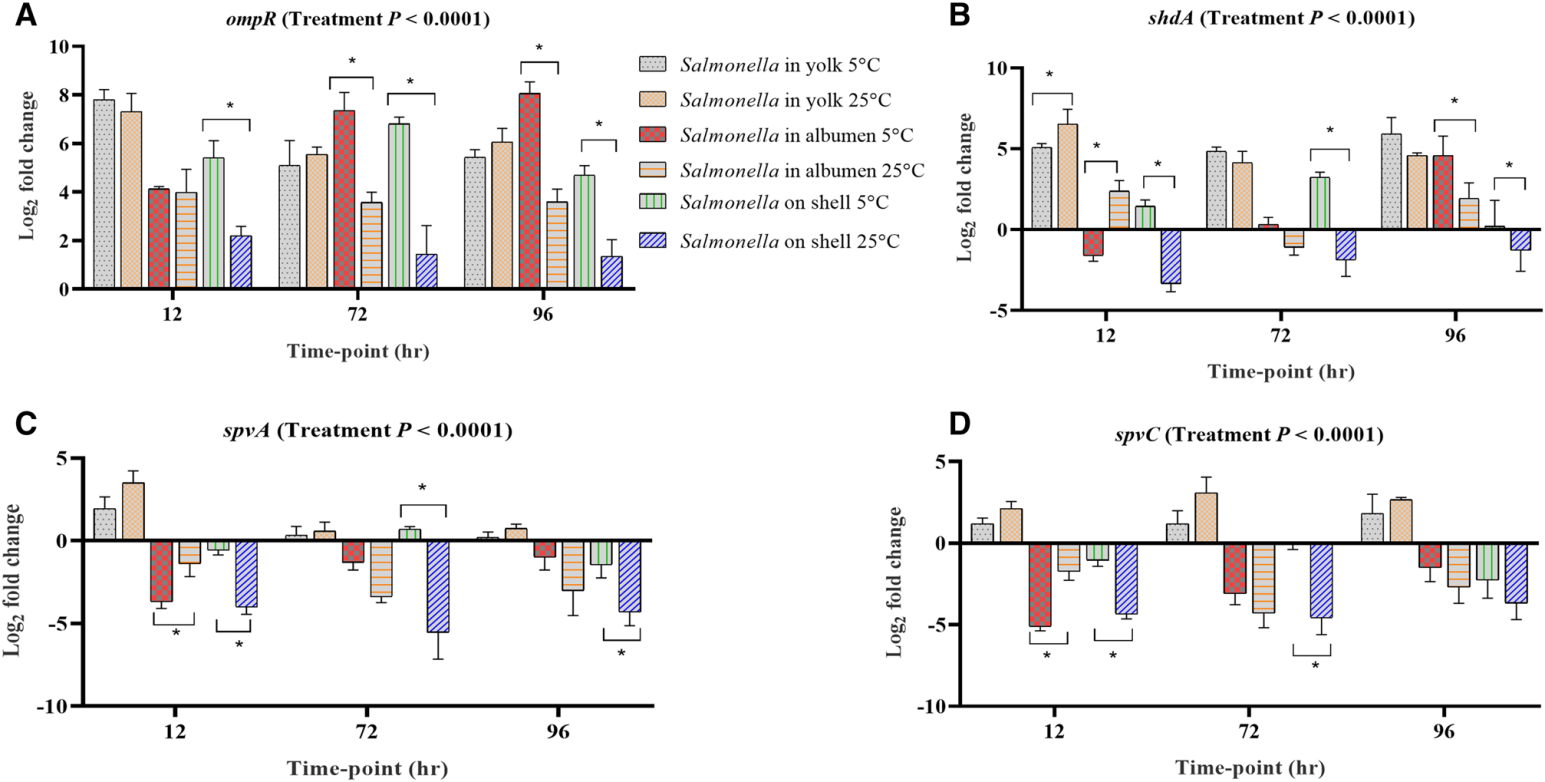
Regulation of genes at different time-points of *Salmonella* inoculated yolk, albumen and eggshell stored at 5 °C and 25 °C. Relative expression (log_2_ fold change ± S.E.) of (**A**) *ompR*; (**B**) *shdA*; (**C**) *spvA* and (**D**) *spvC*. Within each egg component and storage time-point, asterisks across the bars show significant differences (P < 0.05).

Among the genes involved in stress response, *ompR* was the only up-regulated gene across all the samples at the stored conditions (Fig. 4A). For albumen and egg surface samples, the mean fold change of *ompR* was significantly higher at 5 °C compared with the 25 °C stored samples. *shdA* was not consistently regulated in albumen and on egg surface, while in yolk, it was consistently upregulated both at 5 °C and 25 °C at the storage time-points (Fig. 4B). The expression of *sdhA* is involved in colonization as shown in mice challenged with *Salmonella* Typhimurium^27^. *spvA* and *spvC* were up-regulated in yolk but were down-regulated in albumen and on egg surface stored at 5 °C and 25 °C for 96 h (Fig. 4C,D).

#### Flagella, fimbrae and biofilm formation

**Figure 5:**
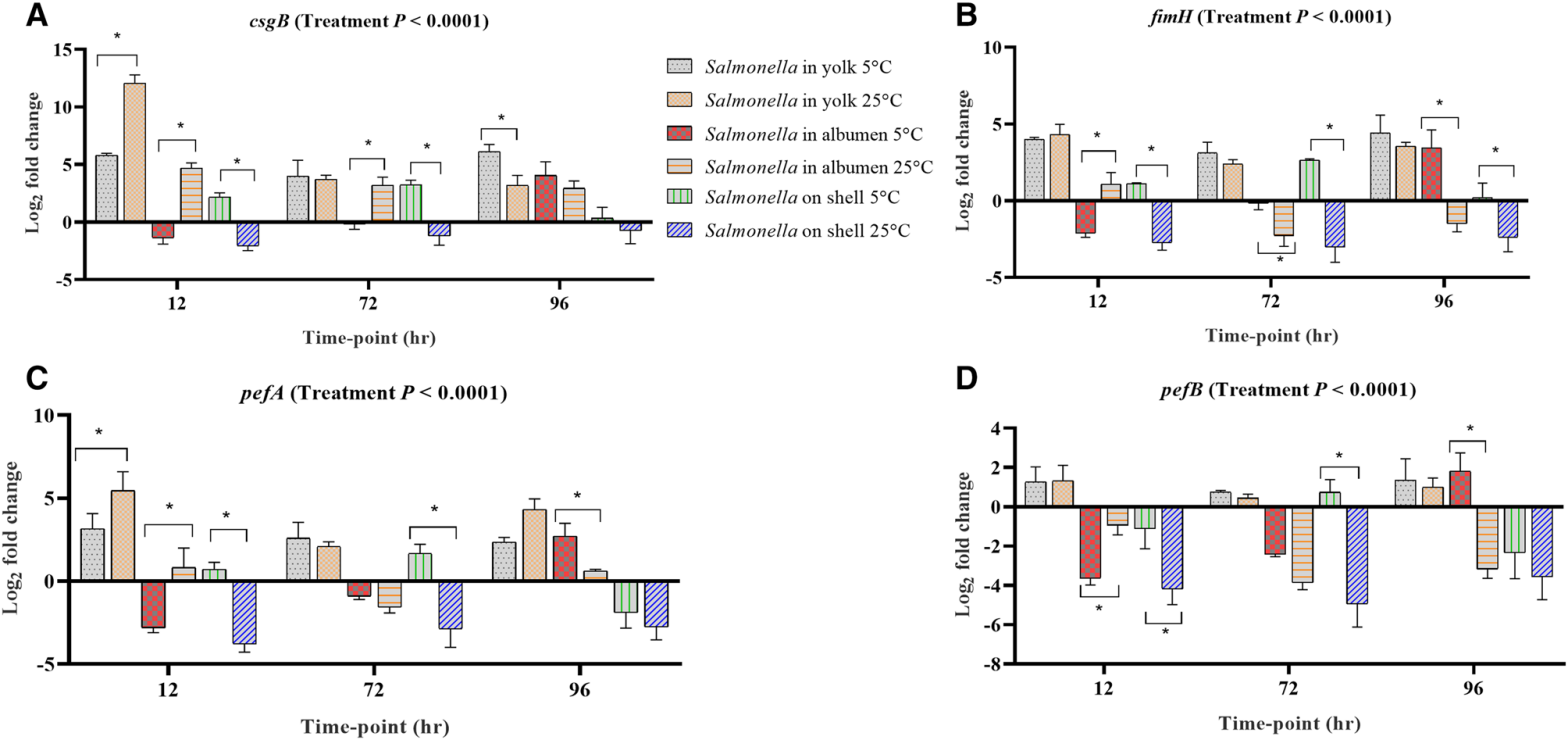
Regulation of genes at different storage time-points of *Salmonella* inoculated yolk, albumen and egg surface stored at 5 °C and 25 °C. Relative expression (log_2_ fold change ± S.E.) of (**A**) *csgB*; (**B**) *fimH*; (**C**) *pefA* and (**D**) *pefB*. Within each egg component and storage time-point, asterisks across the bars show significant differences (P < 0.05).

In biofilm formation, both curli and cellulose synthesis are co-regulated by a complex regulatory network in which *csgD* (*agfD*) acts as a global regulator^28^ in the expression of *csgB* and *csgA*^29^. In the yolk stored both at 5 °C and 25 °C, *csgB*, *fimH*, *pefA* and *pefB* were up-regulated across all the time-points, while their up-regulation was consistent in albumen and on the surface of eggs across the storage time-points (Fig. 5A–D). In the albumen stored at 5 °C, *csgB*, *csgD*, *fimH*, *pefA* were significantly up-regulated at only 96 h. The expression of *bapA* is up-regulated in the formation of curli and cellulose through the involvement of csgD^26^. This indicates that *Salmonella* on the egg surface at 5 °C resisted well to the low temperature. Genes such as *yaiC* (*adrA*), *fimA*, *fliA* and *fliC* were up-regulated at least in one egg component at one storage time-point.

#### Glycolysis, purine and pyrimidine metabolism

**Figure 6:**
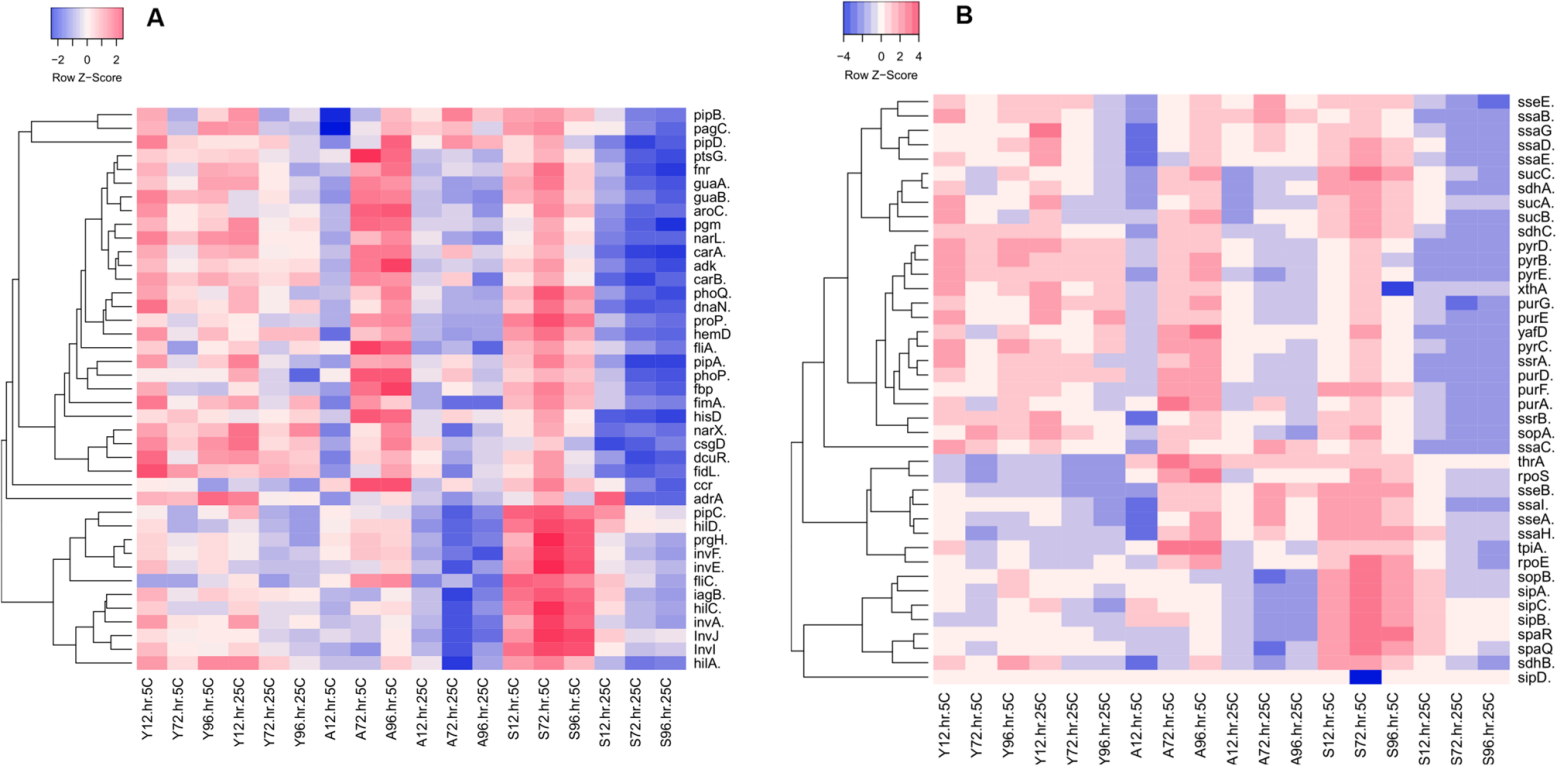
Mean relative expression of genes regulated at two different temperatures (5 °C and 25 °C) and at different storage time-points (12, 72 and 96 h) in various egg components (Y for yolk; A for albumen and S for shell wash). The log_2_ fold change expression values were visualized in Heatmapper web software by using the average linkage clustering method and Spearman rank correlation. Panels (**A**) and (**B**) show the list of 82 genes divided into two groups for the ease of visualization.

Multiple genes involved in cell metabolism were up-regulated in the yolk stored both at 5 °C and 25 °C for 96 h (Fig. 6A,B). Among the investigated genes, *carA*, *carB*, *purD*, *pyrB*, *pyrD* and *pyrE* were up-regulated in the yolk stored both at 5 °C and 25 °C across all the storage time-points, while the up-regulations of *purE*, *purG* and *pyrC* were inconsistent (Fig. 6A,B). Unlike samples stored at 5 °C, there were not many *Salmonella* genes significantly up-regulated in albumen stored at 25 °C. In the albumen stored at 5 °C, *adK* and *yaiC* (*adrA*) were significantly up-regulated at 96 h, while *aroC*, *fbp*, *pgm*, *ptsG*, *purA*, *purD*, *purE*, *purF*, *pyrB*, *pyrC*, *pyrD* and *pyrE* were up-regulated both at 72 and 96 h. At the surface of eggs stored at 5 °C, *carA*, *carB* and *purF* were significantly up-regulated at 12, 72 and 96 h. Apart from the genes involved in purine and pyrimidine metabolism, *aroC* catalyses the pathway of chorismate that serves as the starting substrate in the synthesis of aromatic amino acid biosynthesis. The up-regulation of *aroC* confirms that cell integrity was compromised by the refrigerated temperature in *Salmonella* in albumen and on the egg surface. *hisD* was the only gene up-regulated in yolk, albumen and on the egg surface across all the storage time-points except on the surface of the eggs stored at 25 °C. These data indicate that multiple pathways involved in cell metabolism were activated by *Salmonella* Typhimurium in response to temperature and egg component with pathways being more consistently up-regulated in the yolk samples.

#### Two-component system and Salmonella pathogenicity islands

Our data showed that the *phoP* and *phoQ* involved in the two-component system were significantly down-regulated across all the sampling time-points in egg components, except the albumen stored for 72 and 96 h at 5 °C (Fig. 6A). This positively correlated with the expression of other invasion genes such as *prgH*. All of the investigated genes involved in the regulation of type III secretory systems, such as *ssaB*, *ssaC*, *ssaD*, *ssaE*, *ssaG*, *ssaH*, *ssaI*, *sseA*, *sseB*, *sseE*, *ssrA* and *ssrB* were significantly down-regulated in the egg components stored at 5 °C and 25 °C for 12, 72 and 96 h except the *sseA* that was up-regulated on the egg surface at 5 °C. *hilA*, the transcriptional activator of SPI1 and co-activators such as *hilC* and *hilD* were all down-regulated in the egg components stored for 96 h both at 5 °C and 25 °C.

Other genes that inconsistently upregulated in the yolk, albumen and on egg surface were *proP*, *narL*, *narX*, *rpoS*, *xthA* and *yafD*. In brief, *bapA*, *csgB*, *fimH*, *misL*, *ompR*, *pefA*, *pefB*, *purD*, *pyrD*, *pyrE*, *shdA*, *spvA* and *spvC* consistently up-regulated across all the three storage time-points in the yolk are implicated in maintaining the virulence of *Salmonella* in yolk. Interestingly, these genes were not consistently up-regulated in the albumen and on the egg surface.

#### Salmonella Typhimurium invasiveness in mice and development of clinical disease

The treatment groups that received *Salmonella* inoculated yolk stored at 25 °C for 96 h and the positive control group that received *Salmonella* in LB broth stored at 25 °C for 12 h were culled at day 6 and day 8 post-infection, respectively, due to the development of clinical signs with a score of 5 and above. The percent survival rate of the clinically sick mice was very low compared with all other treatment groups that survived until culled at day 21 p.i. (Supplementary Fig. S2A). There was no significant difference in the percent survivability in the treatment groups that received yolk and LB stored at 25 °C. Compared with day 0 of infection, treatment groups that developed clinical signs after day 3 p.i. had 100% morbidity (Supplementary Fig. S2B). During the mice dissection, gross pathological lesions including the emptied gastrointestinal tract and enlarged liver and spleen were observed in the *Salmonella* in yolk and LB inoculated groups.

#### Fecal shedding profile of Salmonella inoculated mice and Salmonella load in organs

**Figure 7:**
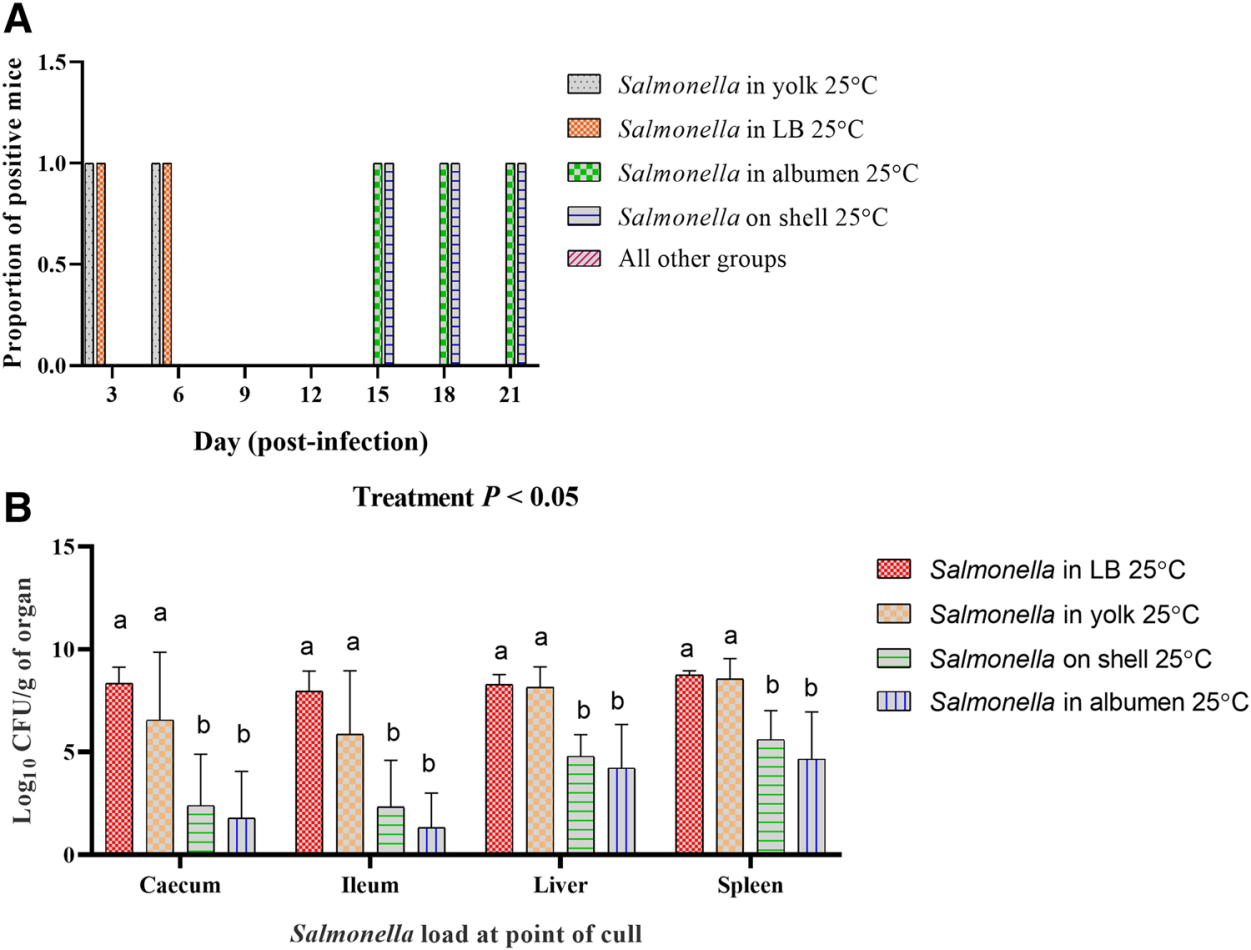
Fecal shedding profile and *Salmonella* load in organs of mice challenged with egg components containing *Salmonella* Typhimurium. (**A**). Qualitative assessment of feces for *Salmonella*. (**B**). Load of *Salmonella* in caecum, ileum, liver and spleen of challenged mice on day of cull. Except of four, the rest of the treatment groups (Table 1) did not shed *Salmonella* in the feces until the termination of the trial on day 21 p.i. Treatment groups that received *Salmonella* in yolk and LB were euthanized on day 6 and day 8 respectively due to the development of clinical salmonellosis. *Salmonella* was not recovered from any of the organs collected from the remaining treatment groups that did not shed *Salmonella* in the feces. Within each organ, different superscripts show significant differences (P < 0.05). In Panel (**A**) of the figure, values are proportion of *Salmonella* positive fecal samples, while in Panel (**B**), values are the mean of log_10_ CFU ± S.D.

Prior to the challenge with *Salmonella* Typhimurium, feces were collected from the mice and tested for the presence of *Salmonella*. All mice were *Salmonella* negative prior to commencing the infection challenge. *Salmonella* was not detected in the feces of control mice or mice infected with egg contents stored at 5 °C at any time-point during the experiment. Feces collected from mice inoculated with LB or yolk stored at 25 °C were positive for *Salmonella* at day 3 and 6 post infection. Mice inoculated with shell wash and egg albumen stored at 25 °C containing *Salmonella* Typhimurium were culture negative for the bacteria in their feces at day 3, 6, 9, and 12 post-infection. At day 15 and 18 post-infection, however, fecal samples collected from mice in these two treatment groups were culture positive for *Salmonella* Typhimurium (Fig. 7A).

*Salmonella* was enumerated from all the organs that were sampled from mice culled during the trial. The organs collected from the treatment groups that did not shed *Salmonella* in the feces were also negative for *Salmonella*, after the enrichment culture method. *Salmonella* load was higher (P < 0.05) in cecum, ileum, liver and spleen of the mice fed with *Salmonella* in LB and yolk compared with the mice received *Salmonella* in albumen and on shell wash stored at 25 °C (Fig. 7B).

## Discussion

The main objective of this study was to understand the temperature driven changes in *Salmonella* Typhimurium inoculated into table eggs. The in-vitro study showed that *Salmonella* Typhimurium grew significantly in yolk by day 4 of storage at 25 °C and from day 4 to day 28, the growth plateaued. A significantly higher load in the albumen inoculated eggs stored at 25 °C on day 7 compared with day 4 showed that *Salmonella* Typhimurium grew in albumen at a slower rate. The slower growth rate of *Salmonella* Typhimurium in albumen compared with yolk could be due to its antimicrobial properties as shown for *Salmonella* Enteritidis^30^. The increased load of *Salmonella* Typhimurium in the yolk of albumen inoculated eggs confirmed that yolk favoured its growth. *Salmonella* Enteritidis has been shown to replicate faster in yolk compared to the albumen of eggs stored at 25 °C^31^. In the albumen inoculated eggs stored at 25 °C, the higher CFUs obtained from yolk further confirmed that *Salmonella* Typhimurium migrated from the albumen and exhibited significant replication.

The *Salmonella* Typhimurium load data in the egg contents indicate that if eggs are stored at ambient temperature, a low load of *Salmonella* can increase in the egg contents significantly. A higher *Salmonella* load on the egg surface at 5 °C compared with the 25 °C confirms that *Salmonella* has the potential to survive on the egg surface at refrigerated temperature and, therefore, it is important to follow proper sanitation guidelines in kitchens while handling eggs. No increase in the load of *Salmonella* in egg albumen and yolk stored at 5 °C supports the notion that eggs should be stored at refrigerated temperature to reduce the growth rate of *Salmonella*. It is important to note that a different inoculum dose was used on egg surface compared to egg internal contents. This was to mimic the field conditions (real-life scenario) where *Salmonella* infected flock can lay eggs with up to 10^6^ CFU/egg. In order to get an inoculum of 10^3^ for mice challenge from the egg surface, egg surface was inoculated with 10^6^ CFU, as eggshells have numerous pores and it is possible that *Salmonella* Typhimurium can penetrate through to pores. Given that *Salmonella* Typhimurium penetration across the eggshell pores was not tested in this study, further investigation is necessary. Overall, the data in the in-vitro whole egg experiment confirmed that within 4 days of inoculation *Salmonella* Typhimurium grew at a faster rate in the yolk of both the yolk and albumen inoculated eggs stored at 25 °C. Therefore, in the subsequent in-vitro and in-vivo experiments, eggs were stored until day 4 post *Salmonella* Typhimurium inoculation.

The Fluidigm PCR data showed that *Salmonella* Typhimurium up-regulated genes involved in cell metabolism, fimbriae formation, stress response, virulence and survival in egg. However, the expression pattern varied with temperature and egg component. An in-vitro study with egg albumen showed that when incubated below 30 °C for a maximum of 24 h, egg albumen exhibited bacteriostatic activity against *Salmonella* Enteritidis^32^. Differences in the survival ability of two genetically similar strains of *Salmonella* Enteritidis in an egg^33^ show that *Salmonella* adopts a wide range of strategies that include nucleotide deletion or insertion. Differences in the gene expression pattern observed in the current study could be due to the variable behaviour of the bacteria, as *Salmonella* Typhimurium expresses its pathogenicity islands differently^34^.

The csg region in *Salmonella* spp., encodes protein polymers known as curli fimbriae, which promote community behaviour and host colonization. In low temperature, curli are important for cell aggregation, adhesion to surfaces and biofilm formation. The up-regulation of *csgB* both at 5 °C and 25 °C in the yolk, albumen and at 5 °C at the egg surface shows that *Salmonella* expressed the curli protein to maintain the physiology of survival and aggregation. Interestingly, in this study, the non-consistent regulation of *csgD* with *csgB* showed their independent regulations in the experimental conditions. yafD in *Salmonella* Enteritidis provides resistance to albumen^22^, and in the current study, a condition dependent up-regulation of expression in the albumen stored at 5 °C for 12 and 72 h was observed. The consistent up-regulation of *ompR* across all the sampling time-points in the egg components stored both at 5 °C and 25 °C shows its role in the survivability of *Salmonella* in an egg. The data showed that *ompR* played a greater role in *Salmonella* survival at 5 °C, as it acts as a central regulator in reprogramming the *Salmonella* transcriptome in a stressful environment^35^. Among the genes involved in osmotic stress response, the up-regulation of *rpoS* both in albumen and yolk at 5 °C shows that *Salmonella* diverted the egg component and temperature driven stress mainly through this gene. However, it is important to note that not all the genes involved in osmotic stress response were included in the current study.

The two-component signal transduction system is involved to modify the cellular output in response to environmental signals both in-vitro and in-vivo conditions^36,37^. The down-regulation of *phoP* and *phoQ* in the egg components show that *Salmonella* was unable to up-regulate its two-component system in the conditions applied in this study. The current study confirms that *Salmonella’s* persistence in yolk, albumen and on the egg surface leads to the downregulation of type III secretory system. Overall, *Salmonella* Typhimurium regulated its transcriptional machinery differently in eggs stored at 5 °C and 25 °C, whereas the temperature driven changes affected the in-vivo virulence capacity of *Salmonella* in the murine model.

The findings in the in-vitro egg studies correlated with the in-vivo mice trial, where the data demonstrated that *Salmonella* Typhimurium in the yolk stored at 25 °C showed the invasive potential that resulted in the development of clinical salmonellosis. *Salmonella* load in the 25 °C stored albumen slightly increased by day 4 of storage; however, the infected mice treatment group that received 10^2^ CFU in albumen did not start shedding *Salmonella* in feces until day 15 p.i. The yolk contains high levels of iron, which is an important requirement for bacterial growth^38^. Growth of *Salmonella* Typhimurium has been shown to increase in response to the presence of iron, and adhesion of *Salmonella* Typhimurium to epithelial cells increases when the bacteria are pre-incubated in higher iron environments^38^. Therefore, this study confirmed that egg yolk can become a higher risk food product if eggs are not stored at refrigeration temperature. Unlike yolk, albumen contains a considerably lower level of free iron^39^. The antimicrobial functions of the main components of fresh albumen, such as lysozyme, ovalbumin, ovostatin, avidin and ovotransferrin depend upon storage temperature and time, and the functions decline faster in eggs stored at 37 °C compared with 20 °C and 4 °C as shown in *Salmonella* inoculated eggs^40^. In the current study, no significant increase in *Salmonella* growth in albumen until day 4 post-inoculation could be due to the antimicrobial function of some of these proteins.

In the current study, the significantly lower percent survival rate, 100% morbidity and higher *Salmonella* load in cecum, ileum, liver and spleen of the 25 °C stored yolk and LB infected mice proved that yolk increased the invasive capacity of *Salmonella* that resulted in the development of salmonellosis. Additionally, the quantitative differences in *Salmonella* load in egg components stored at 5 °C and 25 °C might be contributing factors to the development of salmonellosis in mice. The culling of the sick mice on days 6 and 8 p.i. indicated that although the mice received *Salmonella* through yolk and albumen, *Salmonella* in the yolk resulted in fecal shedding from day 3, while the latter group did not shed the bacteria until day 15 p.i. Shell wash at 25 °C and albumen at 25 °C inoculated mice began shedding *Salmonella* from day 15 p.i. with no clinical signs of salmonellosis during the trial period. This delay in shedding could be attributed to the *Salmonella* being initially stressed due to the storage environment (shell wash and fresh egg albumen). The stress of the treatment group environment (shell wash and albumen) coupled with the exposure to the stressful environment of the digestive tract of the mice^41^ could have caused a delay in the development of salmonellosis and fecal shedding. Although *Salmonella* survived in the egg components at 5 °C storage, the inoculum did not result in the fecal shedding of *Salmonella* in mice during the trial period. Surprisingly, this was the case for the *Salmonella* stored LB in the 5 °C treatment group as well. Observing gross pathological lesions during dissection in the *Salmonella* in yolk and LB (stored at 25 °C) inoculated groups confirmed that these mice developed salmonellosis. However, these findings were not observed in the mice from the albumen or shell rinsate inoculated or any other group that did not shed *Salmonella* in the feces. In Australia, upon collection from the layer sheds, eggs are stored at temperatures $\le$ 15 °C on farms. This temperature is then maintained during grading steps at farms. Further studies are necessary to investigate the effect of farm storage temperature on *Salmonella* virulence.

## Conclusions

Overall, the growth and survivability of *Salmonella* Typhimurium in egg affected by ambient temperature. The temperature influenced the virulence of *Salmonella* Typhimurium and the storage of inoculated eggs at ambient temperature resulted in salmonellosis. In the in-vitro study, the panel of genes assessed for their functions in maintaining the virulence of *Salmonella* showed that genes involved in metabolism, stress response, virulence, and colonization were down-regulated in the albumen and on the egg surface. In the in-vivo experiment, mice infected with egg wash and albumen containing *Salmonella* stored at ambient temperature started shedding *Salmonella* in feces on day 15 p.i. shows that egg components coupled with storage temperature affected the virulence of the bacteria. The data provide evidence that eggs stored at refrigerated temperature significantly reduces the risk of salmonellosis.

## Supplementary Information

Supplementary Information.
