# Supplementary material for: Refrigeration of eggs influences the virulence of Salmonella Typhimurium
Source: Sci Rep. 2021 Sep 9;11:18026. doi: 10.1038/s41598-021-97135-4 (PMC8429434; doi:10.1038/s41598-021-97135-4)
Supplement: Supplementary file 1 — Supplementary Information. [file 41598_2021_97135_MOESM1_ESM.docx]

**Refrigeration of eggs influences the virulence of *Salmonella* Typhimurium**

Samiullah Khan^a,^ Andrea R. McWhorter^a^, Talia S. Moyle^a^ and Kapil K. Chousalkar^a*^

^a^School of Animal and Veterinary Sciences, The University of Adelaide, Roseworthy, South Australia, 5371, Australia

**Supplementary material Text 1. Procedure for PCR used for primer specificity determination.**

PCR mastermix was prepared in a total of 20 μL reaction volume. Briefly, each reaction contained 4 μL of MyRed Taq Buffer (Bioline, Australia), 0.24 μL of MyRed Taq Polymerase (Bioline, Australia), 1 μL of each of the forward and reverse primers, 2 2 μL cDNA template (undiluted) and 9.76 μL of PCR grade water. The PCR cycling conditions were as follows: Initial denaturation at 94°C for 2 min, then 40 cycles of 95°C for 30 sec, annealing at 60°C for 30 sec and extension at 72 °C for 30 sec. All the PCR products were visualised on 2% agarose gel electrophoresis to confirm product specificity.

**Supplementary material Text 2. Fluidigm PCR protocol used in the study.**

The manufacturer’s protocol was used to carry out the Gene Expression Assay (Fluidigm PN 68000088 N1, Appendix D).

20X TaqMan Gene Expression assays (75 rxns, XS, Catalog # 4453320) was pooled to a final concentration of 0.2X each (180 nM) up to a total volume of 200 μL with 10 mM Tris.

Target molecule amplification: 5 μL pre-amplification reactions for each sample was prepared by mixing 1.25 μL of the 0.2X assay pool, 1 μL of PreAmp Master Mix reaction (Fluidigm, PN 100-5580), 1.5 μL of PCR grade water and 1.25 μL of neat cDNA.

The amplification was performed on the BioRad C1000 with the following cycling parameters: Hold at 95°C for 2 mins, 14 cycles of 95°C for 15s and 60°C for 4 mins followed by hold at 4°C. The products where diluted 1:5 by adding 20 μL of 10 mM Tris to each 5 μL reaction. 3 μL of the 20 X of individual assays were prepared for loading onto the 96.96 (PN BMK-M-96.96) Dynamic Array IFCs by diluting with 3 μL of 2X Assay Loading Reagent (Fluidigm, PN 85000735), after priming the IFC as per the Fluidigm protocol.

qPCR reaction on IFC: 3 μL of Quanta PerfecTa® qPCR Fast Mix, low ROX (Quanta Biosciences, PN 95078-02, and 0.3 μL of 20X GE Sample Loading reagent (Fluidigm, PN 85000735) were added to 2.7 μL of preamplified cDNA. 5 μL of each of assay and sample premix were loaded onto the IFCs according to the manufacturer’s instructions (Fluidigm PN 100-2637, PN 68000130). For the 96.96 array, the cycling conditions on the BioMark^TM^ HD were as follows: 70°C for 40 minutes, 60°C for 30 seconds, 98°C for 1 minute, followed by 35 cycles of 97°C for 5 seconds and 60°C for 20 seconds. Additional 2-minute UNG incubation at 45°C was performed prior to the thermal mix for IFCs to prevent carry over contamination. Data were recorded as Ct values by the BioMark^TM^ HD Collection Software for downstream analysis on Fluidigm Real-Time PCR Analysis Software.


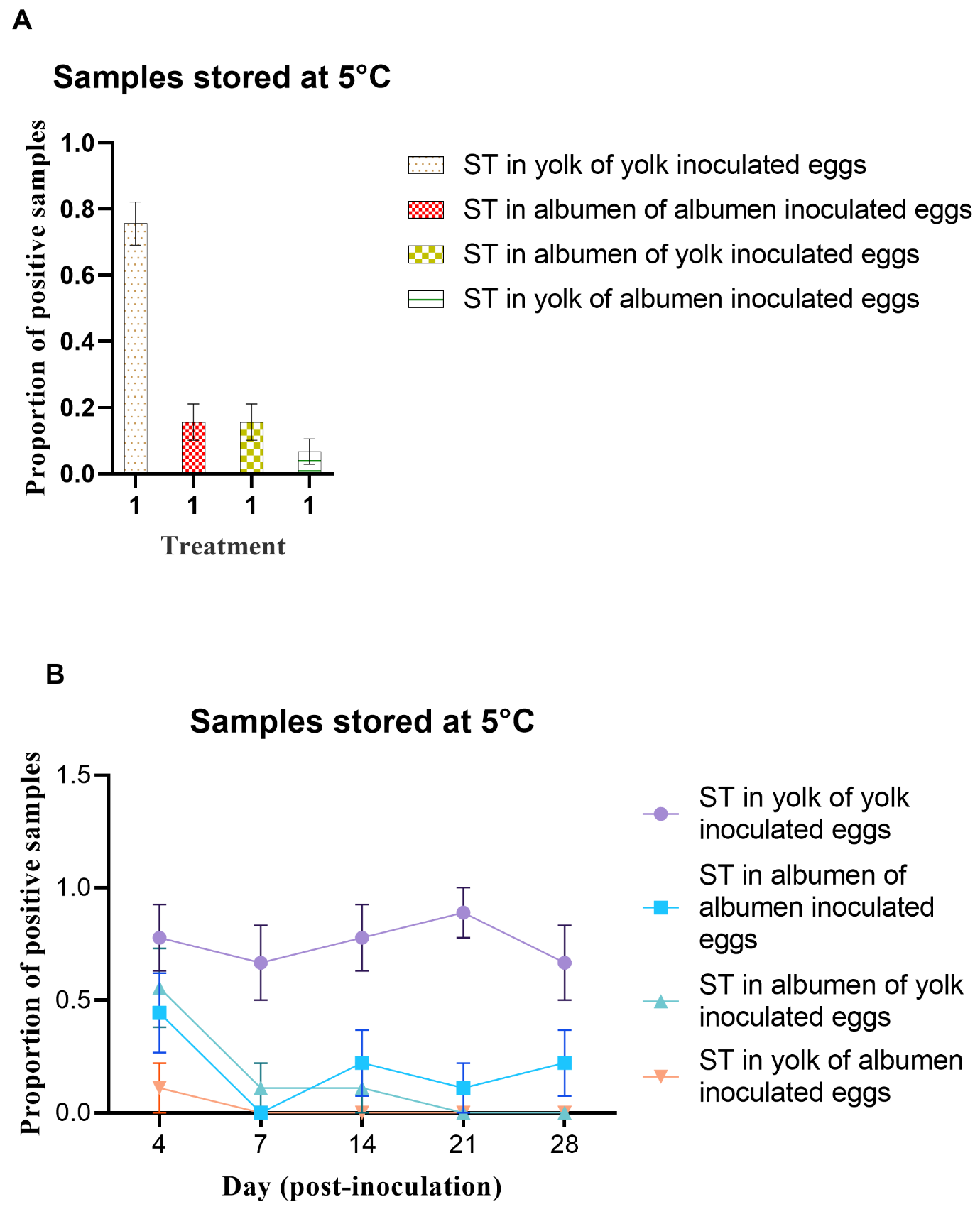


**Supplementary Fig. S1. Proportion of positive samples for *Salmonella* Typhimurium after enrichment in RVS broth.** **A)** Overall proportion of positive samples of yolk and albumen inoculated eggs. **B)** Proportion of positive samples of yolk and albumen at different time-points of storage. Proportion of positive samples was calculated for the 5°C Salmonella inoculated stored eggs. For statistical analysis of the data, Salmonella positive sample was scored as “1” and negative sample was scored as “0”.


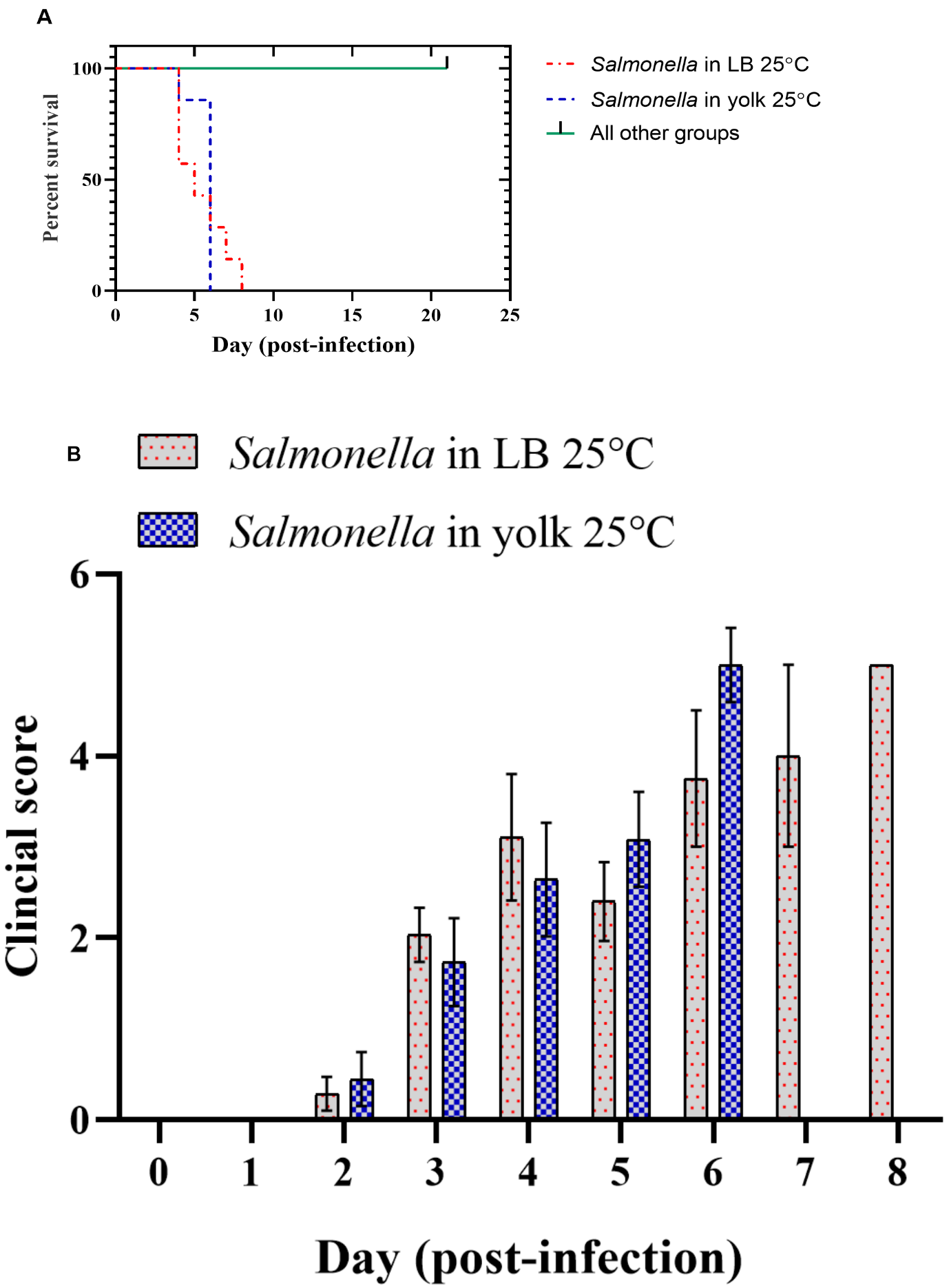


**Supplementary Fig. S2. Percent survival and clinical score of mice infected with *Salmonella* Typhimurium stored in egg components for 96 hr.** **A)** Percent survival. **B)** Clinical score of the two treatment groups showing 100% morbidity rate. The two treatment groups were culled due to the development of the clinical symptoms, while the remaining treatment groups did not develop any clinical symptoms of salmonellosis and therefore survived until the termination of the experiment at day 21 p.i. *Salmonella* in LB stored at 5°C or 25°C for 12 hr were positive control in the study.
